# Spontaneous Perigastric Hematoma and Hemoperitoneum Associated With Rivaroxaban Therapy: A Case Report and Review of the Literature

**DOI:** 10.7759/cureus.116624

**Published:** 2026-09-21

**Authors:** Bouya Rachad, Darius Mbina Nzienghi, Naoufal Chouaib, Ahmed Belkouch

**Affiliations:** 1 Emergency Medicine, Mohammed V Military Teaching Hospital, Rabat, MAR

**Keywords:** conservative management, direct oral anticoagulants (doac), hemoperitoneum, perigastric hematoma, rivaroxaban

## Abstract

Spontaneous hemoperitoneum is a rare but potentially life-threatening complication of direct oral anticoagulant (DOAC) therapy. We report the case of a 59-year-old man receiving long-term rivaroxaban for atrial flutter, previously treated with tricuspid isthmus ablation, who was admitted for atypical chest pain and subsequently developed acute abdominal pain during observation. Contrast-enhanced computed tomography (CT) revealed spontaneous intra-abdominal hemorrhage presenting as a perigastric hematoma with hemoperitoneum, with the perigastric component measuring 96 mm × 58 mm, without active contrast extravasation or an underlying focal lesion. Throughout the admission, the patient remained hemodynamically stable, with no active contrast extravasation on CT and only a moderate decline in hemoglobin levels, from 14.8 g/dL at admission to a nadir of 12.3 g/dL, without requiring transfusion. He was managed with interruption of rivaroxaban and close clinical and biological monitoring alone, with full recovery and no invasive procedure required. A focused review of six comparable published cases of rivaroxaban-associated spontaneous intra-abdominal hemorrhage showed that most required invasive intervention for hemodynamic instability, underscoring the favorable outcome in this hemodynamically stable patient and supporting an individualized, severity-adapted approach to management. The case is notable for the combination of a sizeable perigastric hematoma, moderate hemoperitoneum, absence of active extravasation, hemodynamic stability, and successful non-invasive management. This combination appears to be rarely reported among previously published rivaroxaban-associated intra-abdominal hemorrhages, which predominantly required invasive intervention.

## Introduction

Direct oral anticoagulants (DOACs) have largely replaced vitamin K antagonists for stroke prevention in atrial fibrillation and atrial flutter, owing to their predictable pharmacokinetics and comparable or lower rates of major bleeding [1,2]. Rivaroxaban, an oral factor Xa inhibitor, is among the most widely prescribed DOACs [1]. Despite an overall favorable safety profile, spontaneous intra-abdominal hemorrhage is a recognized, though uncommon, complication. While the overall incidence of major bleeding with DOAC therapy is estimated at 1.1%-4%, spontaneous intra-abdominal hemorrhage specifically has no established incidence rate and is reported almost exclusively through isolated cases [3-7]. Its presentation is often nonspecific, with abdominal pain, unexplained anemia, or hemodynamic instability, and can mimic other acute abdominal emergencies, potentially delaying diagnosis [3]. Contrast-enhanced computed tomography (CT) is central to diagnosis, allowing confirmation of intraperitoneal bleeding, exclusion of an underlying lesion, and identification of active extravasation that may alter management [8]. We report a case of spontaneous perigastric hematoma with hemoperitoneum successfully managed conservatively and review comparable published cases to characterize the spectrum of presentation and management of this entity.

## Case presentation

A 59-year-old man with dilated cardiomyopathy complicated by atrial flutter, previously treated with tricuspid isthmus ablation and maintained on rivaroxaban 20 mg once daily and bisoprolol, with gout treated with febuxostat and benign prostatic hyperplasia treated with an alpha-blocker, was admitted for atypical chest pain. He had been receiving rivaroxaban for at least three years at presentation, with no concomitant use of antiplatelet agents or nonsteroidal anti-inflammatory drugs (NSAIDs). Blood pressure was 137/85 mmHg, heart rate was 120 beats/min, oxygen saturation was 98% on room air, and the Glasgow Coma Scale (GCS) score was 15/15 [9]. A cardiac work-up, including troponin measurement, was initiated. During observation, he developed acute-onset severe abdominal pain, with a tense and diffusely tender abdomen but no guarding or rebound tenderness. There was no history of trauma, fall, or recent invasive or surgical procedure before symptom onset.

Hemoglobin was 14.8 g/dL at admission and declined to a nadir of 12.3 g/dL on day 3, without requiring transfusion. Leukocytosis with neutrophilia and mildly elevated C-reactive protein and potassium levels were observed. Prothrombin time was initially 65% (INR 1.35) at admission and subsequently declined to a nadir of 32% (INR 2.39) during the bleeding episode, consistent with an effect of ongoing rivaroxaban exposure. Activated partial thromboplastin time ratios remained near-normal throughout (1.1-1.2), as expected with a factor Xa inhibitor. The INR was interpreted with caution because it is not a reliable measure of anticoagulation intensity with factor Xa inhibitors. Renal function remained normal throughout the admission, with serum creatinine levels of 11, 10, and 9 mg/L on days 1-3, respectively.

Contrast-enhanced abdominopelvic CT demonstrated spontaneous intra-abdominal hemorrhage presenting as a well-circumscribed perigastric hematoma (Figure 1), measuring 96 mm × 58 mm, with associated moderate hemoperitoneum extending to the right iliac fossa and pelvis. No discrete gastric, omental, mesenteric, or other focal source of bleeding was identified on imaging. A diagnosis of spontaneous perigastric hematoma with hemoperitoneum, most likely associated with rivaroxaban therapy, was considered after exclusion of traumatic, neoplastic, and vascular causes.

**Figure 1:**
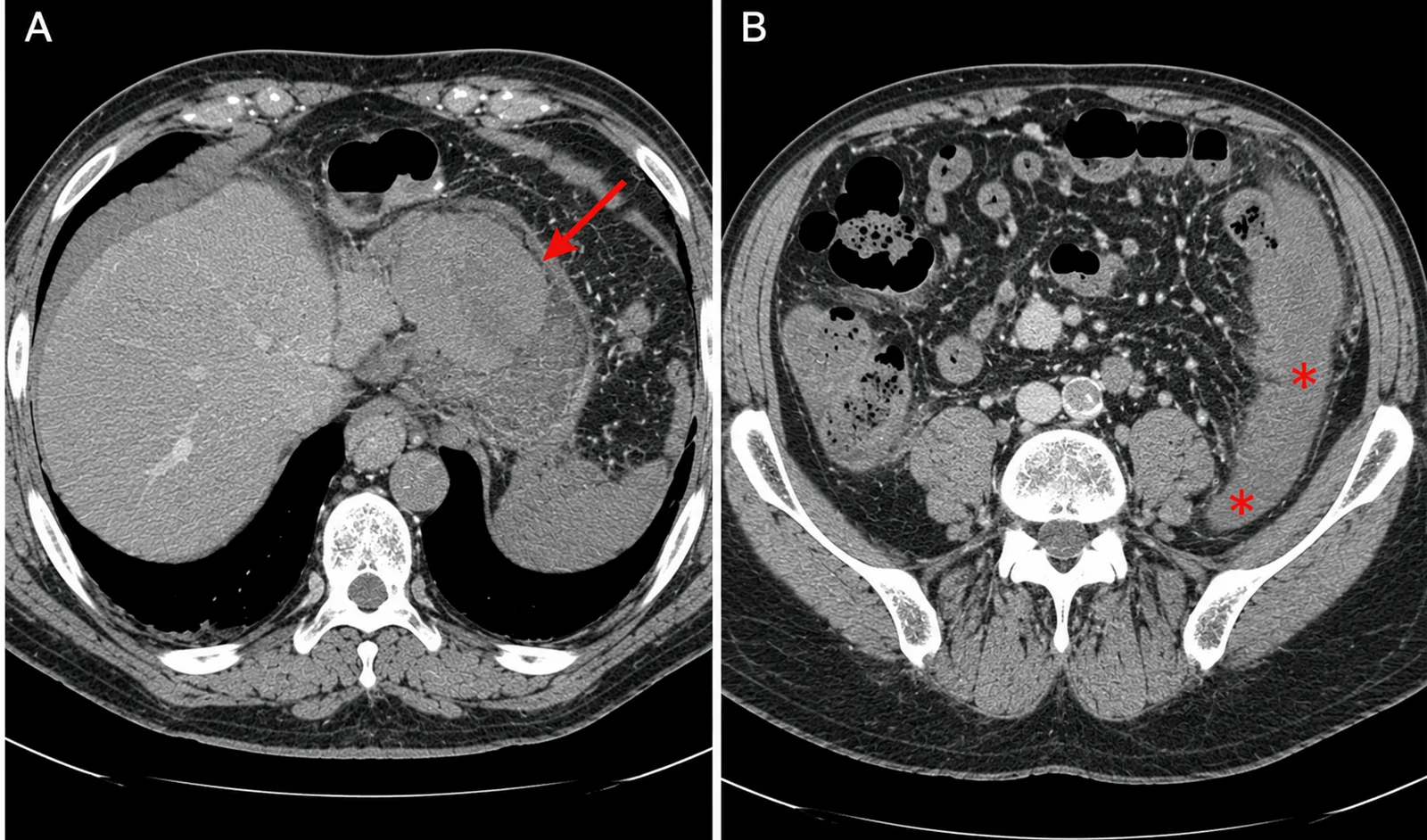
Contrast-enhanced abdominal CT illustrating a spontaneous perigastric hematoma with associated moderate hemoperitoneum in a patient receiving rivaroxaban (A) Axial contrast-enhanced CT image demonstrating a 96 mm × 58 mm hyperattenuating perigastric hematoma (arrow). (B) Axial CT image obtained at a lower abdominal level showing associated moderate hemoperitoneum (red asterisks). No active contrast extravasation or underlying visceral injury was identified, supporting conservative management. CT: computed tomography

Given the patient's hemodynamic stability and the absence of active bleeding on imaging, a conservative strategy was adopted. Rivaroxaban was withheld, and the patient was managed with close clinical and laboratory monitoring alone, without administration of a reversal agent, prothrombin complex concentrate, tranexamic acid, or blood products. The clinical course was favorable, with stabilization of hemoglobin levels, resolution of abdominal pain, and no further evidence of bleeding. Rivaroxaban was resumed 72 hours after admission following multidisciplinary reassessment, and the patient was discharged on day 3 without complications.

Structured follow-up beyond hospital discharge was not available for this patient; therefore, the durability of the favorable outcome and the absence of recurrent bleeding or thromboembolic events after discharge could not be confirmed.

## Discussion

Spontaneous hemoperitoneum under DOAC therapy is thought to result from excessive factor Xa inhibition impairing thrombin generation and clot stabilization, such that trivial or subclinical injury to small, low-pressure vessels fails to seal spontaneously and progresses to an overt hematoma [3,8]. The perigastric region is supplied by a dense network of short gastric and gastroepiploic vessels within the gastrosplenic and gastrocolic ligaments, which may be particularly vulnerable to this mechanism. The absence of active contrast extravasation or a pseudoaneurysm on CT in our patient supports a self-limited, probably venous or capillary source rather than a high-pressure arterial bleed, which is consistent with the favorable clinical course achieved under conservative management alone.

Comparison with the literature

Table 1 summarizes six comparable published cases of rivaroxaban-associated spontaneous intra-abdominal hemorrhage identified in a focused literature search. Most involved atraumatic splenic rupture, with hepatic subcapsular hemorrhage and gallbladder perforation reported less frequently; the majority of these patients developed hemodynamic instability requiring emergency splenectomy, cholecystectomy, massive transfusion, or transcatheter arterial embolization. In contrast, our patient remained hemodynamically stable throughout, despite a sizeable 96 mm × 58 mm hematoma, allowing successful non-operative management without transfusion, reversal therapy, or any invasive procedure. This observation broadens the currently recognized spectrum of rivaroxaban-associated spontaneous intra-abdominal bleeding: five of the six comparator cases required invasive management for hemodynamic instability, while one (Nagaraja et al.) was managed with embolization without a clearly reported instability status [10-16]. This supports the feasibility of individualized, severity-adapted decision-making rather than management dictated by the anatomical site or estimated volume of hemoperitoneum alone.

**Table 1:** Published cases of rivaroxaban-associated spontaneous intra-abdominal hemorrhage NR: not reported

Author	Year	Age/sex	Bleeding site	Hemodynamic status	Indication for anticoagulation	Management	Transfusion/reversal agent use	Outcome
Gonzva et al. [10]	2014	65/NR	Splenic rupture	Unstable	NR	Emergency splenectomy	NR	Recovery
Amin et al. [11]	2016	NR	Splenic rupture	Hemorrhagic shock	NR	Splenectomy	NR	Recovery
Nagaraja et al. [12]	2018	77/F	Splenic rupture	NR	NR	Splenic artery embolization	NR	Recovery
Kwok et al. [13]	2018	80/M	Gallbladder perforation with hemoperitoneum	Unstable	Atrial fibrillation	Cholecystectomy	Blood products administered; coagulopathy correction attempted	Recovery
Labaki and De Kock [14]	2022	64/M	Splenic rupture	Shock	Atrial flutter (recent ablation)	Massive transfusion+splenectomy	Massive blood transfusion	Recovery
Waters et al. [15]	2025	78/F	Hepatic rupture with hemoperitoneum	Shock	Atrial fibrillation	Hepatic artery embolization	Blood transfusion (Hb nadir 6.4 g/dL)	Recovery
Present case	2023	59/M	Perigastric hematoma with moderate hemoperitoneum	Stable	Atrial flutter (previously treated by ablation)	Conservative management only	None (no transfusion, no reversal agent)	Recovery at discharge

Role of imaging

Contrast-enhanced CT is both diagnostic and instrumental in guiding this decision. Beyond confirming intraperitoneal bleeding, it localizes and quantifies any associated hematoma, excludes an underlying structural, neoplastic, or vascular lesion that would otherwise change management, and identifies active contrast extravasation or a pseudoaneurysm when present, arguably the single strongest predictor supporting non-operative management in appropriately selected patients [8]. In our case, CT excluded any focal hepatic, splenic, pancreatic, or appendicular abnormality, reinforcing a purely anticoagulant-related mechanism rather than an occult secondary cause.

A focused, non-systematic literature search was conducted in PubMed/MEDLINE using the terms “rivaroxaban,” “spontaneous,” “hemoperitoneum,” “intra-abdominal hemorrhage,” and “splenic rupture,” restricted to case reports published in English between 2016 and 2025. Cases were considered comparable if they described spontaneous, non-traumatic intra-abdominal hemorrhage temporally associated with rivaroxaban therapy in the absence of an identified structural lesion.

It should be noted that the comparator cases involve different anatomical bleeding sites (spleen, liver, and gallbladder) rather than the perigastric location observed in our patient, which may limit direct comparability despite the shared underlying mechanism.

Management principles

In hemodynamically stable patients without active extravasation, conservative management (anticoagulant withdrawal, correction of hemostasis, and close clinical and laboratory monitoring) is often sufficient, reserving transcatheter embolization or emergency surgery for hemodynamic instability or evidence of ongoing bleeding, as required in most of the comparator cases in Table 1 [3,10,13,16]. Specific factor Xa inhibitor reversal with andexanet alfa was not required in our patient; although effective, its use in routine practice remains limited by cost, restricted availability in many health systems, and reports of thrombotic events following administration. Four-factor prothrombin complex concentrate, while not a licensed specific antidote, remains a pragmatic and more widely available alternative for managing factor Xa inhibitor-related bleeding when needed [15,16].

Resumption of anticoagulation

This decision must balance the thromboembolic risk of the underlying arrhythmia against the risk of recurrent hemorrhage. In our patient, the CHA₂DS₂-VASc score was 1 (attributable to LV dysfunction, with an LVEF <50% on echocardiography), reflecting a modest but non-negligible thromboembolic risk in the setting of atrial flutter previously treated with ablation. The decision to resume rivaroxaban at 72 hours, earlier than the seven- to 14-day intervals typically reported in observational cohorts of anticoagulation resumption after major bleeding, was based on hemoglobin stabilization from a nadir of 12.3 g/dL over the preceding 24-48 hours, absence of active extravasation on imaging, and multidisciplinary consensus balancing thromboembolic and hemorrhagic risk in this individual patient [17-19]. Observational evidence in patients resuming anticoagulation after major extracranial bleeding, including gastrointestinal bleeding and, in a related population, intracranial hemorrhage, consistently shows a lower incidence of thromboembolic events and all-cause mortality once effective hemostasis is achieved, without a substantial increase in recurrent bleeding in appropriately selected patients [17-19]. This supports an individualized approach based on dynamic clinical reassessment rather than a fixed time interval, although the certainty of this evidence remains limited by the observational nature of the underlying studies.

Limitations

As with most reports of this kind, our observation is based on a single patient and cannot establish causality between rivaroxaban and the hematoma beyond a temporal and mechanistic association; an occult contributing vascular fragility cannot be entirely excluded despite a thorough imaging work-up. Longer follow-up would also have provided further reassurance regarding the durability of this favorable outcome after anticoagulation resumption.

## Conclusions

Spontaneous perigastric hematoma with hemoperitoneum is a rare but clinically important complication of rivaroxaban therapy that should be considered in anticoagulated patients with unexplained acute abdominal pain, even in the absence of trauma or hemodynamic instability. Contrast-enhanced CT is essential for diagnosis and therapeutic planning. In hemodynamically stable patients without active extravasation, conservative management can achieve favorable outcomes without invasive intervention, while decisions regarding anticoagulation resumption should be individualized, balancing thromboembolic and hemorrhagic risks.
